# Investigation of a typhoid fever epidemic in Moyale Sub-County, Kenya, 2014–2015

**DOI:** 10.1186/s41043-018-0144-2

**Published:** 2018-05-15

**Authors:** Dahabo Adi Galgallo, Zeinab Gura Roka, Waqo G. Boru, Khalumi Abill, James Ransom

**Affiliations:** 1 Moyale Sub-County Hospital, Moyale, Marsabit County Kenya; 2 Field Epidemiology and Laboratory Training Program, Ministry of Health, Nairobi, Kenya; 3 Piret Partners Consulting, Washington DC, USA

**Keywords:** Typhoid epidemic, Kenya, Sanitation, Laboratory

## Abstract

**Aim:**

Typhoid fever is a vaccine-preventable bacterial disease that causes significant morbidity and mortality throughout Africa. This paper describes an upsurge of typhoid fever cases in Moyale Sub-County (MSC), Kenya, 2014–2015.

**Methods:**

We conducted active hospital and health facility surveillance and laboratory and antimicrobial sensitivity testing for all patients presenting with headache, fever, stomach pains, diarrhea, or constipation at five MSC health facilities between December 2014 and January 2015. We also conducted direct observation of the residential areas of the suspected cases to assess potential environmental exposures and transmission mechanisms. Demographic, clinical, and laboratory data were entered into, and descriptive statistics were calculated with, MS Excel.

**Results:**

A total of 317 patients were included in the study, with mean age 24 ± 8.1 years, and 51% female. Of the 317 suspect cases, 155 (49%) were positive by Widal antigen reaction test. A total of 188 (59%) specimens were subjected to culture and sensitivity testing, with 71 (38%) culture positive and 54 (76%), 43 (60%), and 33 (46%) sensitive to ceftriaxone, cefuroxime, and ciprofloxacin, respectively. Environmental assessments through direct observations showed that commercial and residential areas had limited (1) clean water sources, (2) latrines, and (3) hygiene stations for street food hawkers and their customers.

**Conclusions:**

Typhoid fever is endemic in MSC and causes significant disease across age and sex groups. The local health department should develop policies to (1) assure community access to potable water and hygiene stations and (2) vaccinate specific occupations, such as food and drink handlers, against typhoid.

## Introduction

**Fig. 1:**
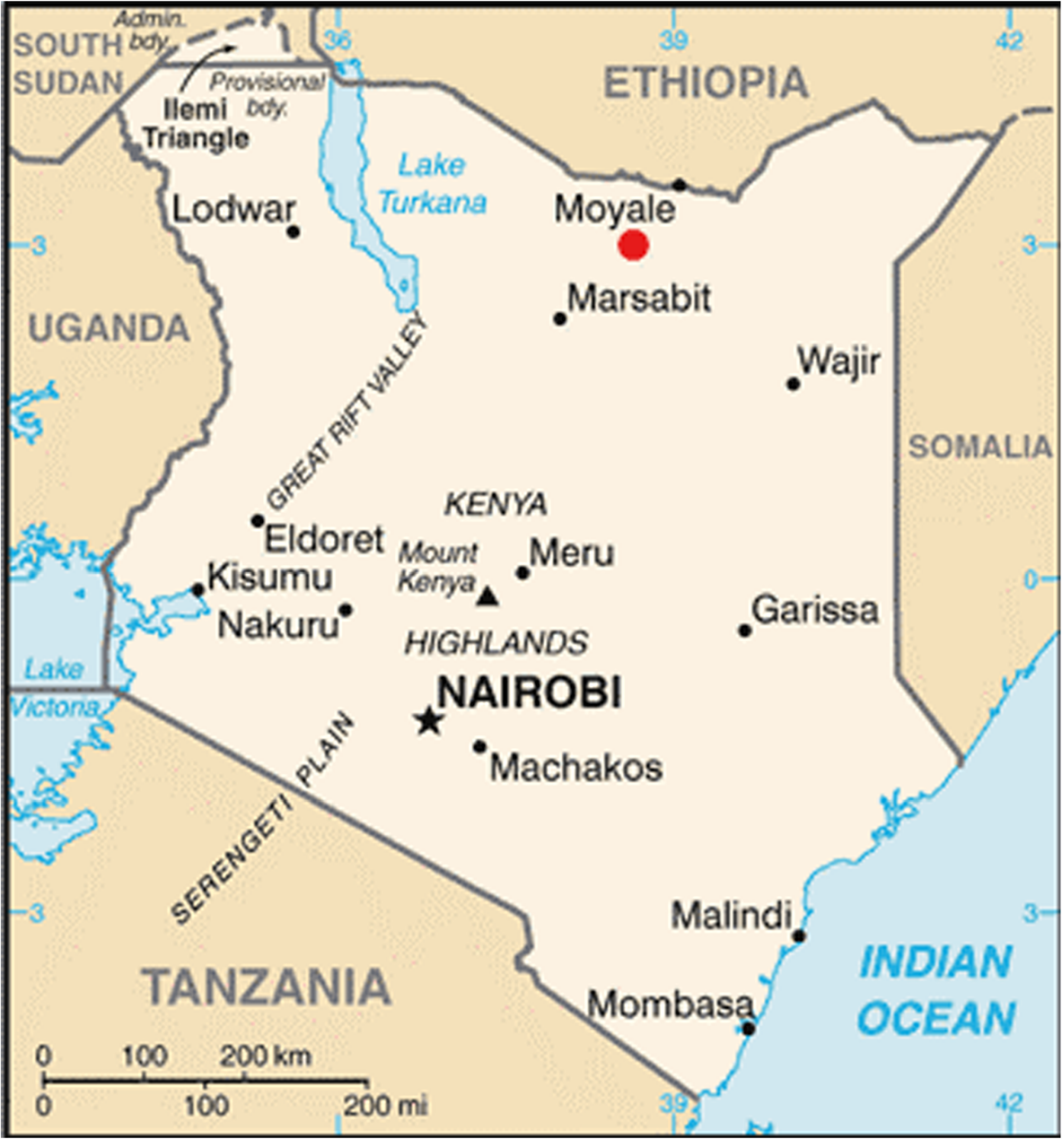
Map of MSC

Typhoid fever, caused by the bacterium *Salmonella* typhi (*S. typhi*), sickens millions of people each year and remains a significant public health problem in low-income countries [1]. Annual incidence in Africa ranges from 13 to 845 cases per 100,000 population, but its epidemiology in Kenya is poorly characterized [2]. In 2014, Moyale Sub-County (MSC), the northernmost point of Marsabit County, reported 3498 cases of typhoid—an annual incidence three times higher than the highest estimates in African countries [1]. MSC (population 130,000) shares a border with Ethiopia and is served by 90 health facilities comprised of 4 hospitals, 17 health centers, 54 dispensaries, 13 private health facilities (nursing homes), and 2 functional clinical laboratories (Fig. 1). This report describes a hospital- and health facility-based epidemiological investigation of suspected typhoid cases at five health facilities in MSC, from December 2014 to January 2015.

## Methods

### Descriptive epidemiology

We conducted active hospital and facility surveillance for clinically diagnosed cases of typhoid fever by reviewing records from five health facilities in MSC of patients admitted between December 5, 2014, and January 5, 2015. Our case definition was any patient presenting with clinical signs and symptoms consistent with typhoid fever, including headache, fever, stomach pains, diarrhea, or chronic constipation. Laboratory procedures included rapid diagnostic tests (Widal), confirmatory culture from stool specimens, and antimicrobial sensitivity testing. Sociodemographic, clinical, and laboratory data were entered into, and descriptive statistics were calculated with, MS Excel.

### Environmental survey

We conducted direct environmental observation and site visits to the most affected areas (based on reported residence of suspect cases) over a consecutive 3-day period. Each day was a 12-h continuous session in areas home to most cases to examine access to hygiene stations (toilets, water) and frequency of handwashing by food and drink vendors and their customers. Documentation included detailed observation notes and photographic evidence of the environment.

## Results

### Descriptive epidemiology

**Fig. 2:**
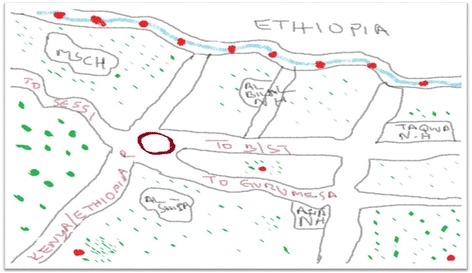
Map of the five health facilities accessed as part of the typhoid epidemic investigation, Moyale Sub-County, Kenya, 2014–2015

A total of 317 patients were identified between December 5, 2014, and January 5, 2015, with mean age of 24 ± 8.1 years. Most [95 (30%)] cases were from Taqwa Nursing Home. MSC Hospital and Al Shifa Clinic had 82 (26%) and 73 (23%) cases, respectively (Fig. 2).

### Laboratory

Of the 317 patients, 155 (49%) were positive via the Widal rapid diagnostic test, with 87 (56%) < 18 years old. Of the 317 patients, 188 (59%) submitted a stool specimen suitable for culture, of which 71 (38%) showed growth. Antimicrobial testing on culture-positive samples showed sensitivity to ceftriaxone [54 (76%)], ciprofloxacin [33 (46%)], and cefuroxime [43 (60%)], which is the drug of choice for treatment of typhoid in MSC (Table 1).

**Table 1:** Patient demographic characteristics and positive typhoid laboratory results, Moyale Sub-County, Kenya, 2014–2015

Variable	*N* (%)
Sex (*n* = 157)	
Male	77 (49)
Female	80 (51)
Age group (years) (*n* = 155)	
< 18	87 (56)
> 18	68 (44)
Facility (*n* = 157)	
Taqwa Nursing Home	47 (30)
Moyale Sub-County Hospital	40 (26)
Al Shifa Clinic	36 (23)
Afya Nursing Home	17 (11)
Al Bilal Nursing Home	16 (10)
Residence (*n* = 155)	
Township	37 (24)
Biashara Street	37 (24)
Sessi	37 (24)
Butiye	22 (14)
Manyatta	17 (11)
Gurumesa	5 (3)
Laboratory	
Widal (*n* = 317)	155 (49)
Culture (*n* = 188)	71 (38)
Antimicrobial sensitivity testing (*n* = 71)	
Ceftriaxone	54 (76)
Cefuroxime	43 (60)
Ciprofloxacin	33 (46)
Ofloxacin	27 (38)
Nitrofurantoin	25 (35)
Chlorophenicol	23 (32)
Gentamicin	19 (26)
Amoxicillin	11 (15)

Any record that did not contain the specific part of the case’s sociodemographic information was excluded from the analysis; therefore, denominators differ from one variable to the next

### Environmental assessment

**Fig. 3:**
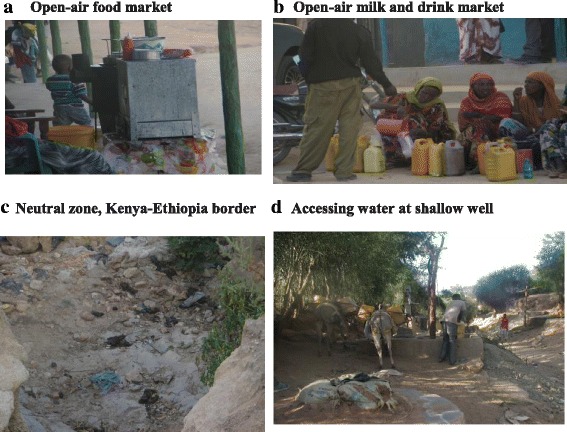
**a**–**d** Potential sources of spread documented during direct observation/environmental assessments, typhoid epidemic investigation, Moyale Sub-County, Kenya, 2014–2015

We observed multiple open-air food and drink markets in many of the areas where the cases reside (Fig. 3a, b). Within the city, there is a no-man’s land between the Kenya and Ethiopia sides of MSC where animal and human waste are deposited very near a water source (Fig. 3c). Villages such as Manyatta and Butiye, which reported the fewest number of positive cases, depend on water from the main water supply or sometimes from harvested rain water. Analysis of the direct observation notes also showed that people in areas that reported most of the positive cases depend on water from shallow wells, which are likely contaminated with human waste (Fig. 3d).

During the 96 h of observation, 55% (65/119) of vendors’ and 69% (37/54) of customers’ fecal-hand contamination events were not followed within 15 min by handwashing. Potential transmission of fecal material to food, drink, or mouth occurred in 64% of cases within 1 h of hand contamination. When we measured the amount of water used at these sites, the mean water usage (6.5 l) was low compared to international disaster relief standards [3].

## Discussion

A typhoid epidemic was detected in MSC due to an increase in the numbers of Widal tests performed at the MSC Hospital laboratory in 2014. This epidemic, which may be part of an overall increase in prolonged, severe, and widespread typhoid outbreaks in both rural and urban populations in Africa, seemed to be predicated on limited access to and use of safe water and sanitation [4]. One possible explanation for the low use of water and poor hand hygiene practices, especially in areas with many commercial food and drink vendors, is the lack of regular access to chlorination products [5]. In low-income countries like Kenya, typhoid infection is usually acquired by consumption of food or water contaminated with human excreta that contains *S. typhi* [1].

Our results via direct observation of the cases’ environments highlight the need for more effective interventions to improve knowledge of typhoid fever transmission and prevention and increase uptake of preventive behaviors such as handwashing [6, 7]. Areas experiencing inaccessibility to clean and safe water like Sessi, Township, and Biashara Street had the most cases (111 [71%]), whereas areas with access to clean water like Butiye, Manyatta, and Gurumesa had the fewest cases (44 [28%]). Areas where food hawking was rampant, such as in Township and Biashara Street, had more cases of people who were < 18 years of age, possibly due to more frequent visits to the food and drink vendors.

In our study, the antimicrobial sensitivity results and the overall culture results are suspect because of rampant self-medication with antibiotics that can be purchased at pharmacies throughout MSC. It is probable, from the histories provided by the patients, that they had antibiotic treatment prior to coming to hospital, hence complicating our ability to interpret culture results [8].

Despite better surveillance and understanding of the epidemiology of typhoid fever, public health seems to be losing the battle against typhoid fever in most African countries [9]. Traditional “hygiene” recommendations did not prevent this epidemic, as there was no mechanism for the communities to remediate the water that was available to them nor to install proper latrines near their homes or near the market areas. Because necessary improvements to water and sanitation are likely to take decades to achieve, we think that targeted and appropriate vaccination campaigns or availability of typhoid vaccines at health facilities could potentially reduce morbidity and mortality [10]. Recommendations should also focus on targeting water treatment packs to the areas that have the bulk of cases, which would reduce risk of specific practices rather than discuss elimination, which is not practical in most affected areas.

## Abbreviations

MSC: Moyale Sub-County
*S. typhi*: *Salmonella* typhi

## References

[1] Mogasale V, Maskery B, Ochiai RL, Lee JS, Mogasale VV, Ramani E, Kim YE, Park JK, Wierzba TF (2014). Burden of typhoid fever in low-income and middle-income countries: a systematic, literature-based update with risk-factor adjustment. Lancet Glob Health.

[2] Wirth T (2015). Massive lineage replacements and cryptic outbreaks of Salmonella typhi in eastern and southern Africa. Nat Genet.

[3] Obani P, Gupta J (2016). Human security and access to water, sanitation, and hygiene: exploring the drivers and nexus.

[4] Bennett SD, Lowther SA, Chingoli F, Chilima B, Kabuluzi S, Ayers TL, Warne TA, Mintz E (2018). Assessment of water, sanitation and hygiene interventions in response to an outbreak of typhoid fever in Neno District. Malawi PloS one.

[5] Branz A, Levine M, Lehmann L, Bastable A, Ali SI, Kadir K, Yates T, Bloom D, Lantagne D (2017). Chlorination of drinking water in emergencies: a review of knowledge to develop recommendations for implementation and research needed. Waterlines.

[6] Alba S, Bakker MI, Hatta M, Scheelbeek PF, Dwiyanti R, Usman R, Sultan AR, Sabir M, Tandirogang N, Amir M, Yasir Y (2016). Risk factors of typhoid infection in the Indonesian archipelago. PLoS One.

[7] Ali E, Van Den Bergh R, D’hondt R, Kuma-Kuma D, De Weggheleire A, Baudot Y, Lambert V, Hunter P, Zachariah R, Maes P. Localised transmission hotspots of a typhoid fever outbreak in the Democratic Republic of Congo. Pan Afr Med J. 2017;28(1):1–9. 10.11604/pamj.2017.28.179.10208 PMC5847255 29541325

[8] Kajeguka DC, Moses E. Self-medication practices and predictors for self-medication with antibiotics and antimalarials among community in Mbeya City, Tanzania. Tanzania J Health Res. 2017;19(4):1–10

[9] Antillón M, Warren JL, Crawford FW, Weinberger DM, Kürüm E, Pak GD, Marks F, Pitzer VE (2017). The burden of typhoid fever in low-and middle-income countries: a meta-regression approach. PLoS Negl Trop Dis.

[10] Walldorf JA, Date KA, Sreenivasan N, Harris JB, Hyde TB (2017). Lessons learned from emergency response vaccination efforts for cholera, typhoid, yellow fever, and Ebola. Emerg Infect Dis.

